# Deciphering Cell-Type-Specific Transcriptional Regulation in Tomato Leaves Through Ensemble Machine Learning and Single-Cell Transcriptomics

**DOI:** 10.3390/plants15101578

**Published:** 2026-05-21

**Authors:** Hui Shen, Wen Liu, Yuanheng Li, Zhaoyilan He, Zheng’an Yang, Zongli Hu, Ting Wu

**Affiliations:** 1 Key Laboratory of Vegetable Biology of Yunnan Province, College of Landscape and Horticulture, Yunnan Agricultural University, No. 452, Fengyuan Road, Panlong District, Kunming 650201, China; hbshenhui@163.com (H.S.); wenliu2021202095@163.com (W.L.); lyhooo0713@163.com (Y.L.); 17787987782@163.com (Z.H.); yangzhengan@ynau.edu.cn (Z.Y.); 2 Laboratory of Molecular Biology of Tomato, Bioengineering College, Chongqing University, Chongqing 400030, China; huzongli71@163.com

**Keywords:** tomato leaf, single-cell RNA sequencing, transcription factors (TFs), hdWGCNA, machine learning

## Abstract

High-throughput single-cell RNA sequencing (scRNA-seq) has substantially advanced plant transcriptional landscapes. However, decoding cell-type-specific transcriptional regulation in non-model crops like tomato (*Solanum lycopersicum*) remains challenging. An integrated computational pipeline was applied using high-dimensional weighted gene co-expression (hdWGCNA) and ensemble machine learning to analyze tomato leaf single-cell transcriptomes. Unsupervised clustering identified 19 cell subpopulations mapped to five major cell-types: mesophyll cells (50.6%), guard cells (31.0%), trichomes (8.3%), vascular cells (7.5%), and lamina epidermis (2.6%). hdWGCNA revealed eight cell-type-specific modules, linking mesophyll cells to photosynthesis and guard cells to redox homeostasis. Machine learning classifiers prioritized candidate transcription factors (TFs), with XGBoost achieving the highest accuracy (0.85) to define cell identity. A consensus of 33 core TFs was identified, from which four candidate TFs (*SlWRKY-78*, *SlWRKY-75*, *SlERF-57*, and *SlGLK-49*) were selected for in silico knockout (KO) analysis. The simulations predicted that these knockouts might dysregulate core functional pathways, such as serine-type endopeptidase inhibitor activity and protein binding. Furthermore, CellOracle simulations suggested that the virtual deletion of the guard-cell-associated *SlWRKY-78* and *SlWRKY-75* could induce a directional trajectory shift from the terminally differentiated guard cells back to the less differentiated mesophyll territory. These findings provide a promising computational framework for deciphering cell-type-specific regulatory programs in horticultural crops.

## 1. Introduction

Tomato (*Solanum lycopersicum*) is a globally cultivated economic crop and a pivotal model species for investigating plant developmental biology, fleshy fruit physiology, and environmental stress responses [1,2]. As the primary organ for photosynthesis, transpiration, and environmental sensing, the leaf plays a foundational role in determining plant architecture, biomass accumulation, and ultimately, agricultural yield [3]. The morphogenesis and functional differentiation of tomato leaves, ranging from the specification of stomatal guard cells to the maturation of photosynthetic mesophyll cells, involve highly coordinated and spatiotemporally dynamic gene expression programs. These intricate cellular processes are strictly governed by transcription factors (TFs), which act as master regulators to orchestrate downstream gene networks [4,5].

Although high-throughput single-cell RNA sequencing (scRNA-seq) technologies have greatly enhanced plant cellular heterogeneity and transcriptional landscapes, computational omics and single-cell resolution studies in tomato remain comparatively underdeveloped relative to model organisms like *Arabidopsis thaliana* [6,7,8,9]. Recently, Yue et al. (2024) established a single-cell transcriptome landscape of healthy and tomato chlorosis virus-infected tomato leaves, providing valuable insights into cellular transitions and gene references for plant–virus interactions [10]. While these findings offer a foundational genetic framework, the deeper regulatory dynamics and complex gene networks operating within specific cell types require further exploration using advanced computational approaches.

Transcription factors (TFs) such as the ERF and WRKY families are well-known master regulators that orchestrate complex gene regulatory networks (GRNs) governing plant growth, development, and stress responses [11,12]. While scRNA-seq offers a unique opportunity to map these regulatory interactions at the single-cell level, constructing robust GRNs from highly sparse and noisy single-cell datasets remains a major computational hurdle [13]. Single-method GRN inference often yields a high rate of false positives and varies significantly depending on the underlying algorithms, such as those relying on gradient boosting machine or tensor representations [14]. Traditional bulk RNA-seq-based correlation analyses are insufficient to capture the intricate, non-linear regulatory dynamics occurring within specific cell lineages [15]. In scRNA-seq data, the selection of informative features is critical for resolving cell-type-specific regulatory programs, as the high sparsity and technical dropout rates can obscure subtle transcriptional differences between closely related cell populations [16].

To overcome these limitations and uncover genuine molecular networks, the application of advanced computational methods and ensemble machine learning algorithms has become indispensable. Integrating diverse computational models, including high-dimensional weighted gene co-expression network analysis (hdWGCNA) for modular visualization alongside sophisticated predictive algorithms like GRNBoost2, scTenifoldNet, and CellOracle, offers a robust strategy to filter technical noise and pinpoint high-confidence regulatory nodes. In this study, we employed an integrated computational pipeline to dissect the TF-mediated regulatory networks in tomato leaves at the single-cell level. By mapping the single-cell transcriptome data and leveraging these machine learning approaches, we identified distinct gene modules and constructed comprehensive regulatory networks to characterize cell-type-specific transcriptional dynamics. This framework not only highlights the precise regulatory hubs of key TFs, such as *SlERF* and *SlWRKY*, but also demonstrates how the integration of advanced computational tools can visualize complex plant molecular networks, bridging the analytical gap in non-model crop systems.

## 2. Materials and Methods

### 2.1. Data Sources

The tomato reference genome assemblies (SL2.5 and SL4.0) were obtained from Sol Genomics Network [17]. Single-cell RNA-seq FASTQ data of healthy tomato leaves (SRX15090984) were retrieved from European Nucleotide Archive (https://www.ebi.ac.uk/ena/browser/view/SRX15090984, accessed on 5 March 2026). The high-confidence and unique marker gene lists of tomato leaf cells were downloaded from PlantscRNAdb [18]. DNase-seq (Sample_01_516, Sample_01_517, Sample_01_522, Sample_01_523, Sample_01_524, Sample_01_561, Sample_01_562, and Sample_01_563) peak sets for tomato leaves were downloaded from the Plant Chromatin Accessibility Database [19]. Gene Ontology (GO) functional annotation datasets for ITAG4.0 were provided by Ricardo et al. on Mendeley Data repository [20]. TF and TF binding motif lists were obtained from MINI-EX [21,22], and matrices of TF binding motifs on the list were sourced from RSAT motif database (https://github.com/rsa-tools/motif_databases, accessed on 5 March 2026) and JASPAR [23].

### 2.2. Process and Basic Analysis of Tomato Leaf scRNA-Seq Fastq Data

The raw scRNA-seq data (SRX15090984) analyzed in this study were obtained from tomato leaves. Healthy leaves were harvested from 30-day-old plants grown under normal conditions. Protoplasts were isolated by enzymatic digestion, and scRNA-seq libraries were constructed using the Chromium Single Cell 3’ GEM Library and Gel Bead Kit v3. Sequencing was performed on an Illumina NovaSeq 6000 platform [10].

Raw sequencing data were processed using the STARsolo pipeline (v2.7.9) to generate a digital gene expression matrix [24,25]. Downstream quality control and data analysis of the raw expression matrix were conducted using the Scanpy computational framework (v1.12.1) in Python (v3.12) [26]. To guarantee the retention of high-quality singlets, cells expressing fewer than 600 or greater than 4000 genes were discarded to exclude dead cells, empty droplets, and potential multiplets. Genes detected in fewer than three cells were also removed from the matrix. Furthermore, Scrublet (v0.2.3) was employed to systematically predict and eliminate homotypic and heterotypic doublets [27]. Feature selection was performed to identify the top 1500 highly variable genes (HVGs) driving transcriptomic heterogeneity. To mitigate the confounding effects of sequencing depth, total counts were regressed out, and expression values were scaled with a maximum threshold of 10. For dimensionality reduction, principal component analysis (PCA) was executed on the HVGs, and the top 20 principal components were utilized to construct a k-nearest neighbors (KNN) graph (k = 15). Unsupervised cell clustering was subsequently performed using the Leiden algorithm at a resolution of 1.2. Furthermore, uniform manifold approximation and projection (UMAP) was applied for non-linear visualization of the cellular landscape [28].

To define the transcriptional signatures of the identified clusters, differential expression analysis was computed utilizing the Wilcoxon rank-sum test on the unscaled, normalized data. Cluster-specific marker genes were strictly defined using a threshold of an adjusted *p*-value < 0.05 and a log2 fold change > 0.25. Cell clusters were subsequently biologically annotated into distinct leaf cell types by intersecting the top defining marker genes of each cluster with a curated tomato leaf marker gene database.

### 2.3. RNA Velocity and Cellular Trajectory Inference

Developmental dynamics and transcriptional transitions of tomato leaf cells were investigated through RNA velocity and trajectory inference analyses. Spliced and unspliced transcript count matrices, previously quantified via STARsolo, were integrated into the annotated single-cell dataset. Following data normalization, the top 2000 HVGs were identified based on the spliced expression layer. First- and second-order moments of expression were then computed across a KNN graph (k = 30) constructed from the top 30 principal components. RNA velocity and cellular latent time were subsequently estimated utilizing Velocity Variational Inference (VeloVI, v0.3.1), a deep generative modeling framework trained on the HVGs to capture complex splicing dynamics [29]. For the reconstruction of continuous developmental trajectories and estimation of cellular plasticity, the CellRank framework (v2.2.0) was employed [30]. Specifically, the CytoTRACE kernel (v1.1.0.4) was applied to infer differentiation potential based on transcriptional complexity [31].

### 2.4. Single-Cell Gene Co-Expression Network Analysis of Tomato TFs

Gene co-expression network analysis at the single-cell resolution was performed using the hdWGCNA R package (v0.4.09) [32]. To construct a biologically robust regulatory network, a custom target gene space was established. Briefly, low-abundance genes expressed in fewer than 0.5% of the total cells were strictly filtered out. All expressed transcription factors (TFs) mapped to the tomato ITAG4.0 annotation (1815 TFs) were retained and combined with the top 1200 HVGs calculated using the “vst” selection method in Seurat (v5.4) [33].

A scale-free topology was achieved by setting a soft-thresholding power of six. A signed topological overlap matrix (TOM) was then constructed. To detect co-expression modules while preserving small, highly specific regulatory clusters, the dynamic tree cut algorithm was applied using optimized parameters: detectCut Height = 0.998, minModuleSize = 30, deepSplit = 3, and a mergeCutHeight of 0.15. Module eigengenes (MEs) were computed to represent the overall expression activity of each module across distinct tomato leaf cell types. Intramodular connectivity (kME) was calculated for all genes within their respective modules using the ModuleConnectivity function. Hub TFs, which likely represent candidate regulators within the network, were defined by ranking the top TFs per module based on their kME scores. To infer the biological significance of the identified modules, GO enrichment analysis was conducted using the clusterProfiler R package, utilizing a customized ITAG4.0 GO annotation database.

To evaluate the temporal dynamics of module activity and hub TFs during cellular transitions, module-specific gene expression patterns were mapped onto inferred pseudotime trajectories. Expression matrices of the top kME-ranked TFs were smoothed across 300 uniform pseudotime bins.

### 2.5. Base GRN Construction of the Prior Gene Regulatory Network

Construction of the foundational prior gene regulatory network (base GRN) necessitated the integration of multi-omics chromatin accessibility data with motif enrichment analysis. Publicly available DNase-seq peak files from tomato leaves, originally annotated under the SL2.5 genome assembly (ITAG 2.4), were aggregated and their genomic coordinates were computationally converted to the SL4.0 reference genome using Burrows-Wheeler Aligner (BWA, v0.7.19) for sequence alignment followed by coordinate liftover [34]. Putative cis-regulatory elements were strictly defined by intersecting these globally accessible genomic loci with targeted promoter regions, spanning 3 kilobase (kb) upstream and 1 kb downstream of the transcription start sites (TSS). The Find Individual Motif Occurrences (FIMO, v5.5.9) algorithm was subsequently deployed to scan the defined accessible promoter sequences for predicted TF binding sites [35]. Finally, the resulting motif–sequence interaction outputs were parsed to construct a binary TF-target association matrix. This matrix was integrated into the CellOracle computational framework (v0.18.0) to instantiate a TFinfo object [36].

### 2.6. Ensemble Machine Learning for Candidate Regulatory Factor Prioritization

To predict candidate TFs potentially associated with cellular identities, we implemented an ensemble machine learning framework utilizing Random Forest (RF) [37], XGBoost [38], and ElasticNet [39]. For the tree-based models, a forest of 1000 estimators was employed; specifically, the RF model used a Gini impurity criterion with max_features set to ‘sqrt’, while the XGBoost model was optimized with a learning rate of 0.05 and a maximum tree depth of six to prevent overfitting. The ElasticNet model, serving as a penalized linear classifier, was tuned using a grid search over an alpha range from 0.01 to 1.0 and an L1-ratio of 0.5 to balance Lasso and Ridge penalties. All models were evaluated via 5-fold stratified cross-validation. TFs were prioritized based on their consensus ranking: feature importance scores were extracted from RF and XGBoost, while absolute coefficient magnitudes were derived from ElasticNet. A high-confidence consensus set was established by extracting the intersection of the top 100 features consistently identified across all three algorithms.

To mitigate algorithmic bias and capture high-fidelity regulatory edges, GRNs were reconstructed using a three-way integration strategy. First, the GRNBoost2 algorithm (within the MINI-EX pipeline) was employed to infer directed regulatory links, from which the top 100,000 edges ranked by importance score were retained. Second, a motif-based GRN was generated using CellOracle, mapping TF-binding motifs to promoter regions within the tomato ITAG4.0 genomic framework. Third, the scTenifoldNet pipeline was utilized to construct a principal component network (PCNet) to capture global gene-gene co-regulation [40]. A set of regulatory interactions was defined as the overarching intersection of the edge lists from these three independent methods. Topological analysis of the set was performed using the igraph and tidygraph packages, where nodes were organized into a three-tiered hierarchy based on their out-degree connectivity. The biological significance of the downstream target genes within this consensus network was further elucidated through GO enrichment analysis.

### 2.7. In Silico Perturbation Simulations and Cell Fate Dynamics

Independent in silico perturbation strategies were deployed to systematically determine the potential regulatory roles of the prioritized candidate TFs across developmental trajectories. We utilized CellOracle for dynamic vector field modeling and scTenifoldKnk for global topological disruption. For trajectory-based simulations within the CellOracle Python framework, the single-cell dataset was initially subsetted to a highly informative feature space. This space comprised motif-annotated TFs, the previously identified hdWGCNA module genes, and the top 2000 HVGs selected via the ‘seurat_v3’ method. KNN imputation (k = 25) was applied within the active principal component space to mitigate single-cell dropout effects. Subsequently, cluster-specific gene regulatory networks were constructed using Ridge regression (α = 1). The expression of individual master regulators was then mathematically suppressed and overexpressed, projecting the resultant shifts in cell state transition probabilities onto the CytoTRACE pseudotime landscape. Finally, local perturbation vectors were aggregated onto a spatial grid (n_grid = 40, smoothing parameter = 1.5, n_propagation = 3), allowing the calculation of inner products to quantify the directionality and magnitude of predicted transcriptional state changes associated with these simulated genetic modifications.

Complementing the trajectory-based predictions, the systemic regulatory footprints of the core TFs were evaluated using the scTenifoldKnk pipeline. Virtual knockouts (KO) for each candidate regulator were simulated within the wild-type (WT) PCNet by mathematically forcing all outgoing regulatory edges from the target node to zero. Non-linear manifold alignment was subsequently utilized to quantify the structural disparities between the WT and the simulated KO networks. Applying a strict significance threshold (FDR < 0.05 and a Z-score magnitude |Z| > 2.0), genes exhibiting substantial regulatory displacement were successfully isolated. Furthermore, gene set enrichment analysis (GSEA) was executed on the complete transcriptome, which was ranked by these regulatory Z-scores via clusterProfiler [41].

## 3. Results

### 3.1. Single-Cell Transcriptomic Heterogeneity, Cell-Type Annotation, and Developmental Dynamics of Tomato Leaves

**Figure 1:**
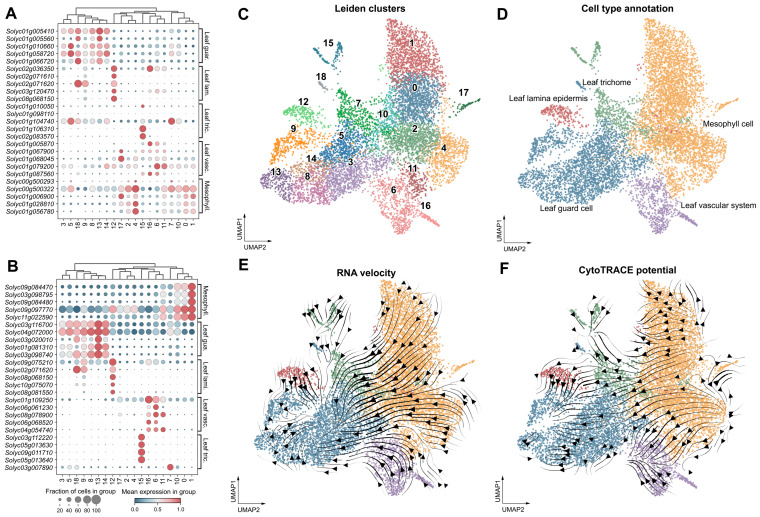
Single-cell transcriptomic landscape, cell-type annotation, and developmental trajectory inference in tomato leaves. (**A**) High-confidence markers (adjusted *p* < 0.05, log_2_ fold change > 0.25). (**B**) Markers with maximal cluster specificity designated as unique. Dot size encodes the proportion of cells expressing a given gene in the cluster; colour intensity reflects the mean expression level of that gene within the corresponding group. (**C**) UMAP embedding of 7993 single-cell transcriptomes, coloured by the 19 Leiden clusters (resolution 1.2). (**D**) UMAP of five annotated cells: mesophyll, guard cells, trichomes, vascular cells, and lamina epidermis. (**E**) Cell-type UMAP overlaid with RNA velocity streamlines inferred by VeloVI. Arrow direction indicates the predicted local transcriptional trajectory; line thickness reflects the magnitude of the velocity vector. (**F**) Cell-type UMAP overlaid with developmental streamlines derived from CytoTRACE. Arrowheads point from regions of high differentiation potential toward regions of low potential; line thickness corresponds to the local velocity magnitude in the CytoTRACE-based transition field.

The initial dataset comprised 9615 cells and 34,075 genes. Sequential removal of low-quality cells, likely multiplets, and lowly expressed genes retained 8703 cells and 23,127 genes. Further exclusion of cells with insufficient spliced and unspliced transcript information yielded a final analytic dataset of 7993 cells. Unsupervised clustering partitioned the cells into 19 transcriptionally distinct clusters, ranging from 1026 cells (12.8%) to 40 cells (0.5%) (Figure 1C). Each cluster was demarcated by a unique set of highly specific marker genes with negligible off-target expression (Figure 1A,B). Representative markers included *Solyc01g107170* for cluster 3 (log_2_ fold change = 2.57, adjusted *p* < 1 × 10^−200^), *Solyc06g072430* for cluster 7 (log_2_FC = 4.00, adjusted *p* < 10^−180^), and *Solyc09g010800* for cluster 15 (log_2_FC = 5.03, adjusted *p* < 10^−80^), with numerous other genes exceeding log_2_ fold changes of 4 at adjusted *p* values approaching machine zero. These sharply defined expression boundaries confirmed that the 19 clusters captured genuine transcriptional heterogeneity in the tomato leaf. Cross-referencing cluster-specific markers with a curated tomato leaf marker database annotated five major cell types (Figure 1D). Each of the 19 Leiden clusters mapped exclusively to a single cell type. The mesophyll constituted the predominant compartment (4047 cells, 50.6%), followed by guard cells (2477, 31.0%), trichomes (661, 8.3%), leaf vascular cells (602, 7.5%), and leaf lamina epidermis (206, 2.6%). On the UMAP, mesophyll cells occupied the broad central and rightward territory, guard cells formed a compact ensemble on the lower left, vascular cells clustered at the bottom right, and trichome and epidermis cells occupied intermediate positions.

RNA velocity streamlines visualised on the UMAP embedding revealed directional transcriptional flux (Figure 1E). The streamlines emanated predominantly from the mesophyll and vascular domains and converged toward the guard cell population, with a secondary branch directed toward trichomes. Inferred latent time positioned guard cells at the most advanced transcriptional state (mean latent time = 2.84), whereas trichomes and vascular cells displayed the lowest mean latent times (1.61 and 1.67, respectively). Mesophyll cells occupied an intermediate latent time (1.76). Trichomes exhibited the greatest average velocity magnitude (0.052), indicative of pronounced transcriptional flux. CytoTRACE analysis provided an orthogonal assessment of differentiation potential (Figure 1F). The CytoTRACE-based transition field, projected as streamlines onto the UMAP embedding, pointed from regions of high differentiation potential toward regions of low potential. The arrow field originated predominantly in mesophyll territory and terminated in guard cell and epidermal regions, with line thickness corresponding to the local transition magnitude. Quantitatively, mesophyll cells harbored the highest developmental potential (mean score 0.68), whereas guard cells scored the lowest (0.33), supporting their annotation as the most terminally differentiated population. Leaf lamina epidermis, vascular, and trichome cells occupied intermediate positions (mean scores 0.58, 0.56, and 0.47, respectively). The CytoTRACE score showed a weak but highly significant negative correlation with latent time (Spearman ρ = −0.25, *p* = 1.9 × 10^−118^), indicating that, although capturing distinct facets of the transcriptome, the two orthogonal approaches suggested on a common developmental trajectory from mesophyll precursors toward terminally differentiated guard cells (Tables S1 and S2).

### 3.2. Weighted Gene Co-Expression Network Analysis Reveals Module-Level Organization and TF-Centric Developmental Dynamics in Tomato Leaf Cells

**Figure 2:**
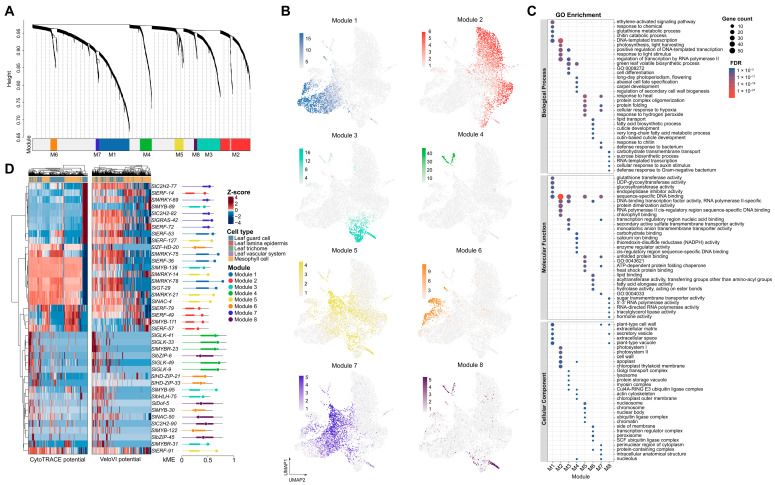
WGCNA reveals cell-type-specific modules enriched for distinct biological functions and hub TFs with dynamic pseudotime trajectories. (**A**) Hierarchical clustering dendrogram of gene co-expression modules identified by hdWGCNA. The eight coloured modules (M1–8) and the unassigned grey module are shown. (**B**) UMAP visualization of module eigengene expression projected onto the 7993-cell tomato leaf transcriptome. Each cell is coloured by the module with the highest eigengene value. (**C**) GO enrichment dot plot for the eight modules. Dot size represents the number of genes in each term, and colour intensity indicates the false discovery rate (FDR). Only the top five terms per module are displayed. (**D**) Heatmap of scaled expression of the top five kME TFs per module along CytoTRACE pseudotime (**left**) and VeloVI latent time (**right**). Rows represent TFs, ordered by hierarchical clustering; columns are ordered by pseudotime bins. The lollipop position shows the TF’s kME value, coloured by module. The black line indicates the highest kME value of the non-TF gene in that module; the thick bar on each lollipop represents the interquartile range of kME for all genes in that module.

Weighted gene co-expression network analysis (hdWGCNA) of the 7993-cell transcriptome identified eight co-expression modules (module 1–8) comprising 2171 genes, with an additional 1013 genes assigned to the unassigned grey module. Module sizes ranged from 31 (module 8) to 303 genes (module 1), and hierarchical clustering of module eigengenes segregated the modules into distinct branches, reflecting divergent transcriptional programs in the tomato leaf (Figure 2A). Visualisation of module eigengene activity on the UMAP embedding revealed sharply defined cell-type-specific expression patterns. Module 1 was predominantly active in guard cells (mean eigengene = 8.82); module 2 characterised the mesophyll (2.64); module 3 showed pronounced specificity for the leaf vascular system (12.37); module 4 and module 5 were largely confined to trichomes (7.68 and 3.15); module 6 was near-exclusively expressed in the leaf lamina epidermis (10.46); module 7 was shared between trichomes and guard cells; and module 8 between vascular and epidermal populations. Each of the 19 Leiden clusters mapped uniquely to a single cel-type, confirming the cell-type-restricted nature of these transcriptional programs (Figure 2B).

Functional annotation by GO enrichment aligned closely with the cell-type assignments. The mesophyll-dominant module 2 strongly enriched for photosynthesis-related processes, including light harvesting (GO:0009765), light stimulus response (GO:0009416), and photosystem I/II components (GO:0009522, GO:0009523). Guard-cell-specific module 1 was characterised by ethylene-activated signalling (GO:0009873), glutathione metabolism (GO:0006749), and glutathione transferase activity (GO:0004364), consistent with hormone-mediated stomatal regulation and redox homeostasis. Module 3 and module 4, associated with the vascular system and trichomes respectively, shared enrichment for green leaf volatile biosynthesis (GO:0010597); module 3 additionally enriched for cell differentiation (GO:0030154) and transcription regulatory region binding (GO:0001067), whereas module 4 was associated with abaxial cell fate specification (GO:0010158) and secondary cell wall biogenesis regulation (GO:2000652). Module 5 and module 7 exhibited overlapping stress-responsive signatures dominated by the heat shock response (GO:0009408), protein folding (GO:0006457), and unfolded protein binding (GO:0051082); module 7 additionally enriched for chitin response (GO:0010200) and bacterial defense (GO:0042742). The epidermis-specific module 6 was uniquely enriched for lipid-related processes central to cuticle formation, including lipid transport (GO:0006869), fatty acid biosynthesis (GO:0006633), cuticle development (GO:0042335), and lipid binding (GO:0008289). Module 8, the smallest module preferentially expressed in vascular cells, showed modest enrichment for carbohydrate transmembrane transport (GO:0034219) and sugar transmembrane transporter activity (GO:0051119), suggesting a role in phloem-associated sugar translocation (Figure 2C; Table S4).

For each module, the top five TFs ranked by kME were examined along the CytoTRACE and VeloVI pseudotime axes in their dominant cell types. In guard-cell module 1, two WRKY TFs (*SlWRKY-78*, kME = 0.76; *SlWRKY-75*, kME = 0.69) exhibited the strongest negative correlations with CytoTRACE pseudotime (ρ = −0.33 each), consistent with declining expression as guard cells mature; a Trihelix (*SlGT-29*) and an ERF (*SlERF-36*) showed weaker but consistent negative trends. Module 4 (trichome) was dominated by four G2-like TFs (*SlGLK-49*, *SlGLK-9*, *SlGLK-33*, and *SlGLK-41*) with the highest mean kME across all modules (0.66) yet negligible pseudotime trends (|ρ| < 0.09), consistent with a role in cell-type maintenance rather than developmental progression. TFs in the other modules generally displayed weak pseudotime correlations in their dominant cell types (|ρ| < 0.3). Notably, several TFs exhibited stronger dynamics in non-dominant populations. Module 1 WRKY and ERF TFs (*SlWRKY-75*, *SlERF-36*) showed strong negative correlations in the vascular system (ρ = −0.66 and −0.44), and module 5 ERF/NAC TFs (*SlERF-91*, *SlNAC-4*) were negatively correlated with pseudotime in vascular and mesophyll cells (ρ = −0.53 and −0.43) despite negligible trends in trichomes. VeloVI correlations were directionally concordant but weaker, and no TF showed opposing trends between the two methods. Across modules, mean kME was highest for module 4 and module 1 (0.66 each) and lowest for module 6 and module 2 (0.30 and 0.37). The 40 top TFs represented 14 families, with ERF (*n* = 9), MYB (*n* = 6), WRKY (*n* = 5), and G2-like (*n* = 4) predominating. ERF and WRKY TFs were broadly distributed across modules 1, 2, 5, and 7, whereas G2-like TFs were exclusive to module 4 and HD-ZIP TFs restricted to the epidermis-associated module 6. In the mesophyll-dominant module 2, five ERF/MYB TFs (*SlERF-14*, *SlMYB-111*) showed consistently positive but weak CytoTRACE correlations, indicative of gradual activation rather than sharp developmental switching (Figure 2D; Tables S5 and S6).

Taken together, these analyses suggest a modular TF architecture in which hub TFs with high kME may largely contribute to cell-type identity maintenance, most notably the guard-cell WRKYs like *SlWRKY-78* and *SlWRKY-75*, additionally correlate with developmental progression. The divergence between intramodular connectivity and pseudotime correlation strength highlights the potential distinction between static module membership and dynamic regulatory activity along the leaf developmental continuum.

### 3.3. Machine Learning Prioritises Candidate TFs Associated with Tomato Leaf Cell Types

**Figure 3:**
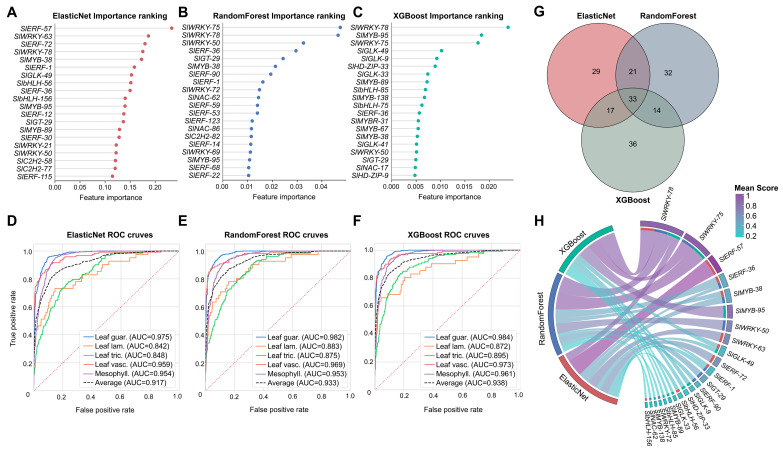
Machine learning identification of candidate TFs associated with tomato leaf cell types. (**A**–**C**) Lollipop plots showing the top 10 TFs ranked by feature importance for ElasticNet, Random Forest, and XGBoost, respectively. TF family is indicated by colour. (**D**–**F**) Receiver operating characteristic (ROC) curves for multi-class classification by ElasticNet, Random Forest, and XGBoost, respectively. Macro-average ROC curves are shown in black dashed lines, and the area under the curve (AUC) is annotated. (**G**) Three-way Venn diagram illustrating the overlap of TFs selected by each algorithm. The central intersection represents TFs identified by all three algorithms, while the pairwise overlaps indicate TFs shared by two of the three methods. (**H**) Circos diagram depicting the relationship between the 10 most important TFs per method and the three machine learning algorithms. The width of each chord is proportional to the normalized feature importance score; the colour of the gene segment reflects the mean importance across methods.

The top 10 TFs ranked by each algorithm were examined as candidate influential features (Figure 3A–C). ElasticNet highlighted ERF and WRKY TFs (*SlERF-57*, coefficient = 0.233; *SlWRKY-63*, 0.187; *SlERF-72, 0.180*; *SlWRKY-78*, 0.175), together with *SlMYB-38* (0.173) and the G2-like TF *SlGLK-49* (0.153). Random Forest assigned the highest importance to WRKY members (*SlWRKY-75*, importance = 0.047; *SlWRKY-78*, 0.047; *SlWRKY-50*, 0.033), followed by *SlERF-36* (0.030) and the Trihelix TF *SlGT-29* (0.024). XGBoost ranked *SlWRKY-78* (0.024) and *SlWRKY-75* (0.018) at the top, alongside *SlMYB-95* (0.018) and a cluster of G2-like TFs (*SlGLK-49*, *SlGLK-9*, and *SlGLK-33*), together with *SlHD-ZIP-33* (0.009), reflecting contributions from epidermis-associated regulators. The recurrent prominence of *SlWRKY-78*, *SlWRKY-75*, *SlERF-57*, and *SlGLK-49* across methods suggested their potential involvement in defining leaf cell-type identity.

The three complementary machine learning algorithms, ElasticNet logistic regression, Random Forest, and XGBoost were trained on the expression matrix of 1109 detected TFs to prioritise those potentially associated with the five tomato leaf cell types. All three classifiers achieved robust overall performance, with XGBoost attaining the highest accuracy (0.85) and macro-averaged F1 score (0.62), followed by ElasticNet (accuracy 0.83, macro-F1 0.67) and Random Forest (accuracy 0.81, macro-F1 0.45). Per-class receiver operating characteristic (ROC) analysis revealed strong discriminative power across all methods (Figure 3D–F). The area under the ROC curve (AUC) values were consistently high for the abundant mesophyll and guard cell populations (AUC > 0.95 across all classifiers) and remained substantial for the rarer cell types. XGBoost yielded the highest average AUC (0.938), with individual class AUCs of 0.984 (leaf guard cells), 0.961 (mesophyll cells), 0.973 (leaf vascular system), 0.895 (leaf trichomes), and 0.872 (leaf lamina epidermis). ElasticNet and Random Forest delivered slightly lower but comparable average AUCs of 0.917 and 0.933, respectively, indicating that TF expression patterns carry sufficient information to computationally resolve leaf cell identity via multiple algorithmic approaches (Figure 3D–F; Table S7).

Intersection of the TF sets selected by each individual algorithm yielded a high-confidence core of 33 TFs that were consistently identified by at least two of the three methods. This consensus set was dominated by several TF families, such as WRKY (*n* = 5), ERF (*n* = 9), MYB (*n* = 5), C2H2 (*n* = 4), and G2-like (*n* = 3) (Figure 3G). Cross-referencing the normalized importance scores of the top 10 TFs with the algorithms that selected them further underscored this convergence further illustrated this convergence. TFs such as *SlWRKY-78* and *SlWRKY-75* were assigned high normalized scores across all three methods in which they appeared (*SlWRKY-78*: ElasticNet 0.38, Random Forest 0.97, XGBoost 1.00; *SlWRKY-75*: Random Forest 1.00, XGBoost 0.64), whereas other TFs such as *SlGLK-49* were prioritized by ElasticNet and XGBoost but not by Random Forest, and *SlMYB-95* was uniquely emphasized by XGBoost (Figure 3H; Table S8).

### 3.4. Integrative GRN Inference and Perturbation Modelling Prioritise Candidate Core Leaf Cell Regulators

The intersection of three putative GRNs (GRNboost2, scTenifoldNet, and CellOracle) retained 536 regulatory edges connecting 72 TFs to 365 target genes, with an average of 7.4 targets per TF. The network was dominated by a small number of highly connected hub TFs, among which *SlWRKY-78* and *SlWRKY-75* were the most prominent, regulating 89 and 75 target genes respectively. Additional hubs included *SlERF-1* (36 targets), *SlWRKY-74* (19 targets), *SlHSF-14* (18 targets), *SlERF-36* (18 targets), and *SlWRKY-72* (17 targets). GRNBoost2 contributed edge weights to all interactions, with the highest confidence scores assigned to edges connecting the major WRKY hubs to their targets, further supporting their centrality in the network. The 72 TFs present in the intersection showed substantial overlap with the 33 core TFs identified by machine learning (Section 3.3), with several of the top ML-ranked TFs also emerging as GRN hubs. This convergence across independent computational approaches supports the prioritisation of these TFs as candidate regulators potentially involved in tomato leaf cell identity (Figure S3; Table S9).

**Figure 4:**
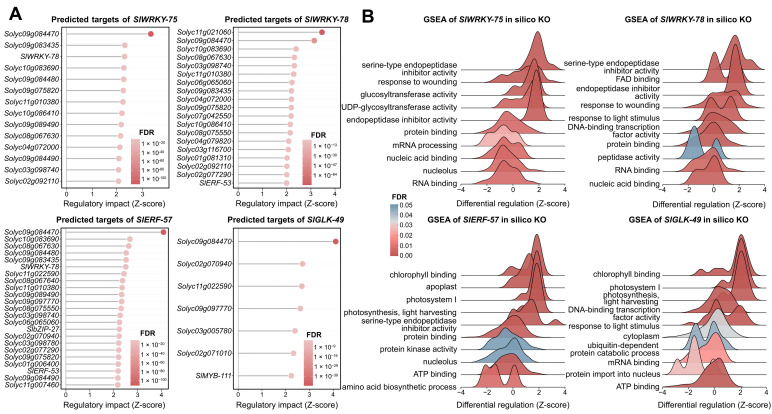
In silico knockout by scTenifoldKnk of four hub TFs reveals predicted target genes and enriched functional pathways. (**A**) Lollipop plots showing the top differentially regulated target genes (|Z|-score ranked, FDR < 0.05) upon virtual knockout of *SlWRKY-78*, *SlWRKY-75*, *SlERF-57*, and *SlGLK-49*. Each point represents a single target gene; the x-axis indicates the Z-score, with positive values reflecting increased target expression upon TF deletion. Point fill colour maps the FDR, with darker red indicating higher significance. (**B**) Ridge plots of GSEA results for the same four knockouts. For each TF, the 10 most significant GO terms, five with the highest positive NES and five with the most negative NES, are displayed. The x-axis shows the Z-score distribution of genes within each term, colour-coded by FDR. The dashed line at Z = 0 separates up- and down-regulated pathway components.

To further interrogate the potential regulatory roles of the four top-ranked hub TFs (*SlWRKY-78*, *SlWRKY-75*, *SlERF-57*, and *SlGLK-49*), in silico knockout simulations were performed using scTenifoldKnk. The number of significantly perturbed genes (FDR < 0.05) ranged from 7 (*SlGLK-49*) to 23 (*SlERF-57*), with Z-scores reaching 4.11 for the most responsive targets. A striking convergence was observed among the two WRKY knockouts, which shared 10 of their differentially expressed targets, including *Solyc09g084470*, *Solyc10g083690*, and *Solyc08g067630*. This extensive overlap suggests that *SlWRKY-78* and *SlWRKY-75* may regulate a highly overlapping downstream programme. Notably, *SlWRKY-78* itself appeared among the top targets of both the *SlWRKY-75* and *SlERF-57* knockouts, further supporting its potential central position within the regulatory hierarchy. Cross-referencing with the 33 machine-learning-derived core TFs revealed that each knockout perturbed one or two core TFs. For instance, *SlERF-53* was affected by *SlWRKY-78* and *SlERF-57*, and *SlMYB-111* was among the *SlGLK-49* targets, suggesting that hub TF knockouts propagate their effects through the core regulatory circuit. One gene, *Solyc09g084470*, was the sole target significantly affected by all four knockouts, highlighting it as a candidate convergent downstream effector of diverse leaf cell-type regulators (Figure 4A).

GSEA of the scTenifoldKnk perturbation profiles was performed on the four hub TFs, and the ten most significant altered GO terms were examined for each knockout. Virtual deletion of all four TFs consistently up-regulated pathways linked to serine-type endopeptidase inhibitor activity (NES = +2.26–2.38) and endopeptidase inhibitor activity (NES = +2.14–2.16 in *SlWRKY-78/-75/-57*), suggesting a shared derepression of protease inhibitor programs. Protein binding was uniformly down-regulated across three of the four knockouts (NES = −1.62, −1.50, and −1.55 for *SlWRKY-78*, *SlWRKY-75*, and *SlERF-57*, respectively), raising the possibility that removal of any single hub TF could broadly affect the protein interaction landscape. RNA binding and nucleic acid binding were additionally repressed in both WRKY knockouts (NES = −1.85 to −1.93), while nucleolus components were down-regulated upon *SlWRKY-75* and *SlERF-57* deletion.

Each knockout also elicited a distinct set of uniquely enriched terms that aligned with its associated cell-type context. *SlWRKY-78* specifically activated FAD binding (NES = +2.23) and light response (NES = +2.11), whereas *SlWRKY-75* uniquely induced glucosyltransferase and UDP-glycosyltransferase activities (NES = +2.21 and +2.20). *SlERF-57* knockout triggered the strongest photosynthetic signature, including chlorophyll binding (NES = +2.51), photosystem I, and light harvesting (NES = +2.41 each), as well as apoplast localization (NES = +2.42). *SlGLK-49*, the sole trichome-associated G2-like factor, prominently up-regulated DNA-binding TF activity (NES = +2.33), the photosynthetic apparatus (photosystem I, light harvesting, chlorophyll binding; NES = +2.42–2.45), and light stimulus response (NES = +2.33), while uniquely down-regulating mRNA binding, protein import into the nucleus, the ubiquitin-dependent proteolytic pathway, and general cytoplasmic components. These divergent signatures indicate that, although the four hub TFs converge on a core stress- and defense-associated transcriptional programme, each may retain distinct functional outputs that reflect its predicted cell-type-specific regulatory role in the tomato leaf (Figure 4B).

**Figure 5:**
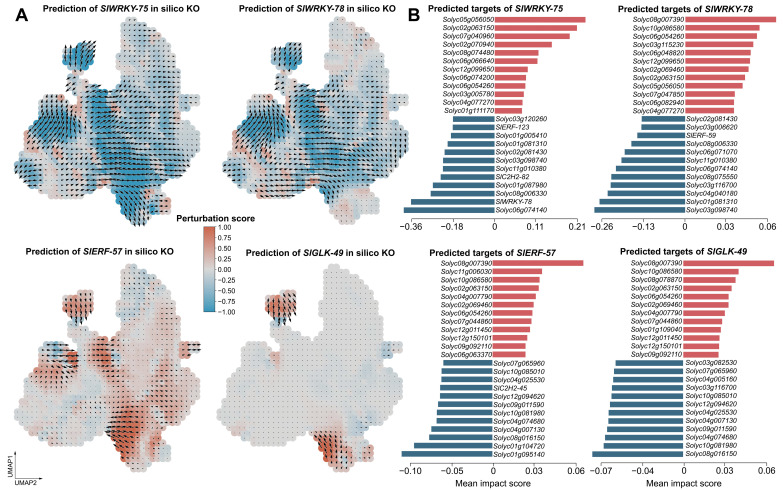
CellOracle perturbation simulations of the four hub TFs. (**A**) UMAP flow fields showing the perturbation score (inner product) upon the KO of *SlWRKY-78*, *SlWRKY-75*, *SlERF-57*, and *SlGLK-49*. Grid colour indicates the perturbation score, with red representing positive scores and blue representing negative scores. Arrows indicate the direction and magnitude of the simulated cell-state shift relative to the CytoTRACE pseudotime gradient. Arrow length indicates the magnitude of the simulated cell-state shift. (**B**) Rank plots of the top 12 genes with the greatest positive (red) and negative (blue) mean expression shift after knockout. Gene labels are displayed at the centre of each plot. The x-axis reports the mean shift score, with actual asymmetric ranges indicated on the axis.

CellOracle perturbation simulations projected the transcriptome-wide effects of each hub TF knockout onto the UMAP embedding, predicting the direction and magnitude of potential cell-state shifts relative to the CytoTRACE pseudotime gradient. Knockout of the two guard-cell-associated WRKY factors, *SlWRKY-78* and *SlWRKY-75*, produced pronounced and highly directional flow fields, with perturbation vectors pointing from the guard cell territory toward the central mesophyll region. This shift is suggestive of a directional transition along the differentiation trajectory, from the terminally differentiated guard cell state toward a transcriptional state reminiscent of less differentiated mesophyll, which is consistent with the strong negative CytoTRACE correlations these WRKYs exhibited in their dominant cell type. In contrast, *SlERF-57* and *SlGLK-49* knockouts generated weaker and more diffuse flow fields, with perturbation vectors distributed across multiple cell types including mesophyll, vasculature, and epidermis. The overall shift direction remained broadly toward the mesophyll center, consistent with a partial loss of differentiation signal, but the reduced magnitude and broader spatial spread align with the more moderate and distributed expression of these TFs across the leaf (Figure 5A).

Consistent with the UMAP flow fields, quantification of the top 12 genes most strongly shifted upon knockout of each hub TF revealed a matching hierarchy of perturbation strength. The two WRKY factors, whose knockout produced the most directional flow toward the mesophyll, also elicited the largest transcriptional changes, with mean expression shifts reaching +0.21 and −0.36 for *SlWRKY-75*. In contrast, *SlERF-57* and *SlGLK-49*, which generated weaker and more diffuse flow fields, showed correspondingly modest gene-level effects, with maximum absolute shifts of 0.10 and 0.07, respectively.

The target gene profiles further mirrored the cell-type specificity observed in the flow fields. The *SlWRKY-78* and *SlWRKY-75* knockouts both shifted cells away from the guard cell territory, shared five down-regulated and five up-regulated targets. Among the repressed genes were the WGCNA-M1 marker *Solyc03g098740* and the transcription factor *SlC2H2-82*, while *Solyc05g056050* and *Solyc02g063150* were among the induced genes. This extensive overlap is consistent with the nearly identical flow patterns of the two WRKY deletions. In contrast, *SlERF-57* and *SlGLK-49*, whose flow fields were spatially diffuse and affected multiple cell types, shared a strikingly concordant target gene profile with one another, comprising six up-regulated and eight down-regulated genes, despite originating from distinct cell-type contexts. Only a small set of genes responded to all four knockouts, highlighting the potential functional diversification among the hub TFs and suggesting the existence of a limited common downstream effector set (Figure 5B).

## 4. Discussion

### 4.1. Comparative Insights into Leaf Cellular Heterogeneity and Developmental Ontogeny

The transition from bulk transcriptomics to single-cell resolution has fundamentally redefined the conceptualization of plant organogenesis. It shifts the perspective from static tissue models to dynamic cellular ecosystems [42]. While the foundational atlas established by Yue et al. (2024) provided a vital baseline for tomato leaf responses to viral infection, the current investigation offers a higher-resolution dissection of healthy leaf architecture [10]. By partitioning the transcriptome into 19 distinct sub-populations, the analysis suggests a finer degree of functional compartmentalization than previously appreciated in horticultural crops. This increased granularity is particularly evident within the mesophyll and vascular lineages. It suggests that tomato leaves, representative of fleshy-fruit-bearing species, possess intricate regulatory layers to manage the high metabolic flux required for both vegetative growth and subsequent reproductive demands [43]. Comparative analysis with model species like *Arabidopsis* reveals both conserved and divergent features in cell-type-specific transcriptomes. The five major lineages, including mesophyll, guard cells, trichomes, vascular cells, and epidermis, align with the fundamental cellular composition of C3 dicot leaves [44,45]. Tomato possesses multicellular glandular trichomes with complex specialized metabolic pathways, in contrast to the unicellular non-glandular trichomes of *Arabidopsis*. This divergence underscores the necessity of crop-specific single-cell models, as the regulatory mechanisms governing trichome development and chemical defense in Solanaceous species cannot be fully extrapolated from the *Arabidopsis* paradigm [46].

Crucially, this analysis delineates a proposed developmental trajectory within the leaf lamina using orthogonal computational metrics. The mesophyll was identified as a putative versatile progenitor state with high differentiation potential, as indicated by a CytoTRACE score of 0.68, and displayed a predicted convergent transcriptomic flow toward terminally differentiated guard cells. These guard cells exhibited features consistent with the highest degree of specialization, marked by high latent time and a low differentiation potential of 0.33. This maturation involves a substantial narrowing to support highly specialized turgor-driven movements. Conversely, the early specification of vascular and trichome lineages contrasts with the protracted maturation of the stomatal lineage, suggesting a temporal separation of cell fate determination. Establishing this high-resolution baseline is essential for deciphering cellular identity, as it provides the foundational context for subsequent ensemble machine learning algorithms to prioritise candidate TFs that may be associated with cell identity programs, distinguishing them from genes that merely serve as downstream markers of established cell states.

### 4.2. Regulatory Logic of Modular TF Networks

The identification of cell-type specific co-expression modules through hdWGCNA provides a systems level framework for understanding the regulatory hierarchy in tomato leaves. This modular organization supports the notion that cellular identity may be governed by coordinated gene suites rather than by isolated marker genes [47,48]. Our findings suggest a significant functional division between cellular lineages. For instance, the transition from photosynthesis-dominated programs in the mesophyll to redox and hormone signaling programs in guard cells reflects the specialized physiological requirements of these tissues. This alignment between modular gene expression and cellular function is consistent with observations from other plant single-cell atlases, where co-expression networks effectively capture the fundamental biological priorities of distinct cell types [49].

A central advancement of this analysis is the delineation of a potential functional dichotomy among hub TFs. While traditional models often emphasize high intramodular connectivity as the primary indicator of regulatory importance, the integration of developmental pseudotime reveals that connectivity and temporal dynamics are often uncoupled [50]. The results suggest that TFs within the tomato leaf operate through two distinct regulatory modes. The first mode involves the continuous maintenance of cellular identity, as exemplified by the G2-like factors in the trichome module. These factors exhibit high connectivity but lack significant temporal trends, suggesting their role is to provide a stable transcriptional environment for specialized metabolic processes. This mirrors the behavior of established identity maintainers in model organisms, where certain TFs are required throughout the life of the cell to prevent lineage reversion [51].

The second regulatory mode is characterized by dynamic tracking of cellular maturation, most prominently observed in the guard cell lineage. The negative correlation of specific WRKY and ERF factors with developmental progression is consistent with these regulators being primarily active during transitional phases. In plant developmental biology, the transition from a progenitor state to a terminally differentiated cell requires rapid transcriptional switches. The current analysis suggests that these dynamic hub factors may function as candidate transitional regulators potentially involved in lineage commitment before being downregulated in mature states. This temporal specificity is often overlooked in static single-cell studies but is essential for accurately modeling the causal drivers of plant organogenesis. By distinguishing between these candidate maintenance and transitional regulators, the current study offers a refined biological context for the subsequent application of ensemble machine learning. Because traditional differential expression analysis often fails to separate primary drivers from downstream targets [52], this modular and temporal framework is required to prioritize TFs with the highest regulatory potential. This systematic approach increases the likelihood that the identified candidates are not merely markers of established cell states; in addition, they represent promising candidates for functional involvement in the underlying regulatory machinery governing horticultural crop development [53].

### 4.3. Integrative Machine Learning and Perturbation Modeling Contextualize Core Regulatory Hubs

The application of ensemble machine learning to single-cell transcriptomics represents a significant methodological advancement over traditional differential expression analysis. The implementation of ElasticNet, Random Forest, and XGBoost successfully reduced the transcriptional complexity of the tomato leaf into a highly predictive core of 33 transcription factors. The robust discriminative power of these models supports the hypothesis that cellular heterogeneity may be governed by a concentrated hierarchy of key regulatory genes. The recurrent identification of G2-like and ERF family members as central regulatory nodes aligns with their widely documented roles in plant development. For example, G2-like factors are classically recognized for regulating chloroplast development, which is consistent with their assignment as stable identity maintainers in photosynthetic tissues [54]. Similarly, the prominent positioning of ERF family members corresponds to their known functions in mediating environmental responses, suggesting that baseline cellular maturation is intrinsically linked to environmental sensing mechanisms [55,56].

The potential regulatory importance of these hub regulators was further interrogated through the in silico perturbation simulations. By deploying predictive modeling approaches, the current investigation transitions from descriptive networks to in silico perturbation-based prioritisation. The simulated knockouts of the guard-cell-specific WRKY factors, notably *SlWRKY-78* and *SlWRKY-75*, induced a highly directional transcriptomic shift toward a less differentiated mesophyll state. This predicted directional shift toward a less differentiated state is consistent with the notion that terminal cellular identity may require active and continuous maintenance by specific hub regulators. The contrasting perturbation flow fields reveal distinct regulatory scales within the leaf. While the WRKY knockouts produced strict lineage reversions, the deletion of factors like *SlGLK-49* resulted in diffuse transcriptomic shifts affecting multiple cell populations, indicating that plant gene regulatory networks operate through varied functional modes.

The computational prioritization of *SlWRKY-78* (homologous to *SlWRKY71*) and *SlWRKY-75* (homologous to *SlWRKY6*) as candidate regulators potentially associated with the guard cell lineage provides a highly resolved cellular perspective on established macroscopic phenotypes. Existing functional characterizations demonstrate that *SlWRKY71* enhances foliar resistance against bacterial pathogens and acts as a transcriptional repressor of suberin deposition in the root exodermis [57,58]. Concurrently, *SlWRKY6* is well documented as a positive regulator of leaf senescence and fruit ripening, with its activity strictly governed by complex post-translational modifications including persulfidation and phosphorylation. While root suberization, global pathogen defense, and macroscopic senescence appear phenotypically distinct from single-cell maturation, they are biologically unified. Guard cells function as the primary physical interfaces for foliar immunity, and their terminal differentiation requires extensive transcriptional reprogramming analogous to the targeted dismantling of baseline programs observed during senescence [59].

Although the specific downstream target genes identified via the in silico perturbation simulations do not exhibit significant overlap with the previously validated targets from bulk stress assays, this divergence is highly informative rather than contradictory [60]. Macroscopic studies typically capture acute transcriptional responses to severe external stimuli or terminal aging across heterogeneous tissue mixtures. In contrast, the single-cell perturbation models simulate the baseline developmental ontogeny of healthy tissue at an isolated cellular resolution [61]. The limited direct target overlap likely reflects the pleiotropic nature of plant transcription factors [62]. These regulators deploy distinct transcriptional sub-networks depending entirely on the immediate cellular context and the specific presence of external signals [42]. Therefore, despite these methodological and contextual differences, the computational results possess substantial predictive potential. They provide a computational framework that supports the potential involvement of established stress responsive regulators as fundamental components of baseline cellular identity, offering a testable hypothesis that integrates cell-type-specific development with broader physiological adaptations [63].

### 4.4. Limitation of This Research

Several methodological limitations should be acknowledged. First, the candidate TFs identified here were prioritized through computational approaches without direct experimental validation. Future in vivo experiments, such as CRISPR-based knockout of the candidate hub TFs followed by single-cell transcriptomic profiling, or targeted RT-qPCR of predicted downstream targets, will be required to confirm whether the transcriptional shifts predicted by scTenifoldKnk and CellOracle are recapitulated in tomato leaves. Whether these in silico predictions fully hold under native cellular conditions remains to be tested. Second, this study was restricted to the transcriptomic level and could not capture post-translational modifications or protein–protein interactions essential for TF activity. Third, standard scRNA-seq requires tissue dissociation, which removes the spatial context needed to map cell types to their exact anatomical positions; spatial transcriptomics could address this in future work [64]. Finally, the virtual perturbation simulations relied on inferred GRNs with inherent false-positive and false-negative rates [65], which could be refined by incorporating single-cell chromatin accessibility data (scATAC-seq), and combined with bulk ATAC-seq [66].

## 5. Conclusions

In this study, we presented a comprehensive computational framework to decipher cell-type-specific transcriptional regulation in tomato leaves using single-cell RNA sequencing, hdWGCNA, and ensemble machine learning algorithms. By analyzing 7993 high-quality single-cell transcriptomes, we successfully annotated five major cell types and traced the developmental dynamics from mesophyll precursors to terminally differentiated guard cells. Through hdWGCNA, we identified module-level organization, linking specific biological functions such as photosynthesis and redox homeostasis to their corresponding cell populations. Furthermore, the integration of multiple machine learning models and network inference identified a consensus set of 33 candidate TFs potentially associated with cell identity. Among them, *SlWRKY-78*, *SlWRKY-75*, *SlERF-57*, and *SlGLK-49* emerged as high-priority candidate regulatory hubs. In silico KO and network perturbation analyses suggested that these potential TFs may contribute to the maintenance of cell identity and the regulation of vital downstream pathways. Specifically, the simulated deletion of guard-cell-associated WRKY TFs induced a directional trajectory shift towards the mesophyll state, consistent with a metabolic shift potentially associated with these selected regulatory genes that may distinguish them from other TFs in shaping guard cell identity. Together, our integrated pipeline offers a promising methodological framework for prioritising candidate regulators understanding cell-type-specific regulatory networks and developmental dynamics in horticultural crops.

## Data Availability

This study analyzed publicly available single-cell RNA sequencing data deposited in the European Nucleotide Archive (ENA) under accession number SRX15090984. All processed data and analysis code generated during this study are included in the article and its Supplementary Materials.

## Supplementary Materials

The following supporting information can be downloaded at https://www.mdpi.com/article/10.3390/plants15101578/s1, Figure S1: Quality control and HVG identification for tomato leaf single-cell RNA sequencing data. (**A**) Scatter plots depicting the relationship between the mean expression of genes and their dispersion, with the left and right panels displaying normalized and raw dispersions, respectively. Black dots represent the selected HVGs, while gray dots denote other genes. (**B**) Violin plots illustrating the distribution of single-cell quality control metrics. The left and right panels show the number of expressed genes and total UMI counts per cell, reflecting sequencing depth and transcript capture efficiency, respectively. Individual cells are represented by the scattered points, which assist in determining appropriate thresholds for filtering out anomalous cells. Figure S2: hdWGCNA soft-thresholding power selection and module eigengene correlation. (**A**) Determination of the soft-thresholding power for network construction. The scatter plots display the scale-free topology model fit (top left), mean connectivity (top right), median connectivity (bottom left), and max connectivity (bottom right) as functions of the soft-thresholding power. The red dashed line indicates a scale-free R^2^ fit of 0.80, and the vertical black dashed line marks the selected soft power threshold (β = 6). (**B**) Correlogram of module eigengenes. The lower triangular matrix shows the Pearson correlation coefficients among the identified gene modules (M1–M8). The color scale from blue to red represents correlations ranging from −1 to 1, where red indicates positive correlation and blue indicates negative correlation. Asterisks indicate statistical significance levels (*, *p* < 0.05; **, *p* < 0.01; ***, *p* < 0.001). Figure S3: Intersection analysis of three GRNs and visualization of the high-confidence network in tomato leaves. (**A**) Venn diagram of inferred gene regulatory networks. The Venn diagram illustrates the overlap of regulatory interactions inferred by three distinct algorithms: GRNBoost2, scTenifoldNet, and CellOracle. (**B**) High-confidence regulatory network. The circular network displays the core interactions shared among the three methods. The central nodes represent key TFs, with node sizes corresponding to the out-degree (number of target genes regulated). The peripheral black dots represent target genes, and edge thickness and coloring represent the interaction scores of the inferred networks. Table S1: Leiden clustering summary and cell-type annotation with top marker genes in the tomato leaf single-cell transcriptome. Table S2: RNA velocity and CytoTRACE developmental metrics across annotated tomato leaf cell types. Table S3: TF list of tomatoes from MINI-EX. Table S4: Top eight GO enrichments of hdWGCNA module genes. Table S5: Pseudotime associations of the top 25 kME TFs per module with CytoTRACE and VeloVI latent time in tomato leaf cell types. Table S6: Gene composition, kME, and TF annotations for all co-expression modules. Table S7: Classification performance and per-class AUC of ElasticNet, Random Forest, and XGBoost. Table S8: Top 10 TFs per algorithm and consensus core set membership. Table S9: GRN intersection of GRNboost2, scTenifoldNet, and CellOracle.
